# Functional Dorsoventral Symmetry in Relation to Lift-Based Swimming in the Ocean Sunfish *Mola mola*

**DOI:** 10.1371/journal.pone.0003446

**Published:** 2008-10-22

**Authors:** Yuuki Watanabe, Katsufumi Sato

**Affiliations:** 1 International Coastal Research Center, Ocean Research Institute, The University of Tokyo, Otsuchi, Iwate, Japan; 2 National Institute of Polar Research, Itabashi, Tokyo, Japan; University of Sheffield, United Kingdom

## Abstract

The largest (up to 2 tons) and a globally distributed teleost—the ocean sunfish *Mola mola*—is commonly regarded as a planktonic fish because of its unusual shape including absence of caudal fin. This common view was recently questioned because the horizontal movements of the ocean sunfish tracked by acoustic telemetry were independent of ocean currents. However, direct information regarding their locomotor performance under natural conditions is still lacking. By using multi-sensor tags, we show that sunfish indeed swam continuously with frequent vertical movements at speeds of 0.4–0.7 m s^–1^, which is similar to the records of other large fishes such as salmons, marlins, and pelagic sharks. The acceleration data revealed that they stroked their dorsal and anal fins synchronously (dominant frequency, 0.3–0.6 Hz) to generate a lift-based thrust, as penguins do using two symmetrical flippers. Morphological studies of sunfish (mass, 2–959 kg) showed that the dorsal and anal fins had similar external (symmetrical shape and identical area) and internal (identical locomotory muscle mass) features; however, the muscle shape differed markedly. We conclude that ocean sunfish have functional dorsoventral symmetry with regards to the non-homologous dorsal and anal fins that act as a pair of vertical hydrofoils. Although sunfish lack a swimbladder, we found that they are neutrally buoyant independent of depth because of their subcutaneous gelatinous tissue that has low density and is incompressible. Efficient lift-based swimming in conjunction with neutral buoyancy enables sunfish to travel long distances both horizontally and vertically.

## Introduction

The largest (up to 2 tons) and a globally distributed teleost—the ocean sunfish *Mola mola*—has a laterally compressed deep body and lacks a caudal fin [1], and appears as if it were “half fish” with the posterior portion cut off [2]. This peculiar appearance gives the impression of a planktonic weak swimmer, compared to pelagic continuous swimmers such as tunas, lamnid sharks, and dolphins. These swimmers have a streamlined body, circular body cross section, narrow caudal peduncle, and caudal fin with a high aspect ratio, which enable them to swim fast and efficiently [3]–[5]. Furthermore, ocean sunfish are often seen lying on their sides and drifting at the sea surface, although they swim slowly in aquariums, apparently by moving their dorsal and anal fins. The common view that ocean sunfish are weak swimmers was recently challenged. Using acoustic telemetry, researchers showed that the ocean sunfish move regardless of the direction of the dominant ocean current, suggesting that they are active swimmers [6]. However, direct information on the locomotor performance of the species is still lacking.

Animal-borne accelerometers have proved to be powerful tools for studying the locomotor behavior of marine animals with limited access. They provide us with important information such as stroking activities and body inclination, and have been extensively applied to free-ranging marine mammals and seabirds [7]. However, studies on fishes with accelerometers are still limited. One practical reason for this may be the fact that accelerometers must be recovered to obtain the data, but the recapture of instrumented fishes is often very difficult. Satellite-linked telemetry is not currently available for acceleration data, which need to be sampled at a high frequency (>10 Hz) and are therefore too large to be sent via satellite. Recently, we solved this problem by developing a time-scheduled release system that allows us to recover the logger without recapture of the instrumented fish [8], [9]. It is now possible to study the swimming performance of ocean sunfish under natural conditions.

Ocean sunfish and their relatives (family, Molidae) have several peculiar features with regard to external and internal morphology that are expected to be related to their swimming behavior. For example, with regard to external features, they lack a caudal fin and have enlarged dorsal and anal fins. Do these non-homologous fins work as a pair of propulsors? With regard to internal features, unlike other fishes in the order Tetraodontiform, Molidae lack a swimbladder [10]. Do they have negative buoyancy and swim continuously to avoid sinking, as is the case in tunas [11]? Alternatively, is their weight in water supported by a low-density liver, as is the case in some sharks [12]? Little is known about the functional morphology in terms of the swimming behavior in ocean sunfish. Generally, literature on ocean sunfish is surprisingly sparse in any area of biology although this species is very popular worldwide. This may be because of their little commercial value and large size, which makes sampling difficult. However, this is not the case in our study area, namely, Otsuchi, Japan. Here, ocean sunfish are commercially caught and consumed, and it is possible to collect samples of ocean sunfish ranging in size from a few kilograms to a ton from fish markets.

In this study, we attached accelerometers to ocean sunfish with a time-scheduled release system to examine their swimming performance under natural conditions. Additionally, we studied the external and internal morphology of sunfish to identify any adaptations for their swimming behavior. Our goal was to understand how ocean sunfish swim on the basis of both behavioral and morphological studies.

## Results

### Behavioral study

**Figure 1:**
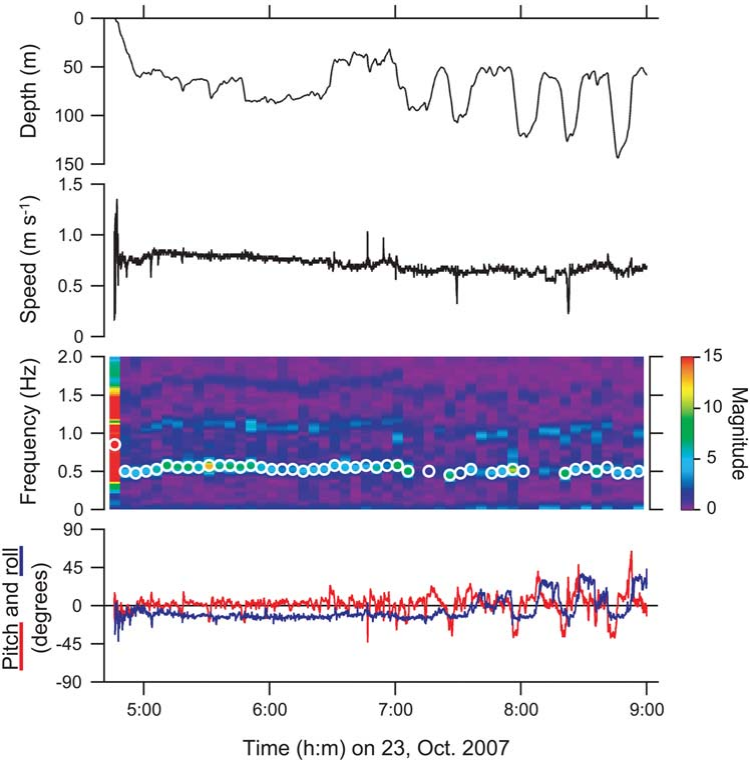
Depth, swimming speed, spectrogram of lateral acceleration (indicative of fin movements) where magnitude was expressed by color, pitch (red line), and roll (blue line) of an ocean sunfish (mass, 48 kg). Open circles on the spectrogram represent dominant stroke cycle frequencies calculated for each five-minute time bin. Positive pitch represents head-up attitude of the fish, while negative pitch indicates head-down attitude. Positive roll represents right-rotated position of the fish, while negative roll indicates left-rotated position.

We recorded behavior of three ocean sunfish (mass, 48, 59, and 153 kg) for 14 h in total (Table 1). According to the recovery points of the loggers, all fish appeared to swim offshore from Otsuchi Bay, which opens to the east, in similar directions (80–89°) at horizontal speeds of 1.5–2.2 km h^−1^. All fish changed their swimming speeds widely during the first few minutes of the records, possibly due to handling. Then, the fish cruised with frequent vertical movements (Fig. 1). Fin movements were continuous throughout the records, and the frequency was constant within individuals regardless of swimming depth and whether the fish was ascending or descending. Pitch of the fish was close to horizontal when swimming horizontally, head-up (maximum, 65°) when ascending, and head-down (minimum, −55°) when descending. Absolute value of roll remained <30° over 80% of the tag records, and exceeded 30° most commonly during horizontal swimming. In one instance, a fish (mass, 59 kg) accelerated for 15 s up to 2.4 m s^−1^ horizontally near the surface with a right-rotated position (roll, 48°) and a high stroke cycle frequency (2.0 Hz). During the recordings, no fish drifted on its side at the surface.

**Table 1:** Swimming behavior of ocean sunfish.

Individual	Total	Body	Data	Swimming		Swimming		Mean	Dominant	Vertical movements >2 m				
	length	mass	length	depth (m)		speed (m s^−1^)		Reynolds	stroke cycle	N	depth change (m)		duration (min)	
	(m)	(kg)	(h)	mean	maximum	mean	maximum	number	frequency (Hz)		mean (±SD)	maximum	mean (±SD)	maximum
Mola07A	0.97	48	4.2	70.1	144.0	0.7	1.0	6.1×10^5^	0.56	90	13.2±19.2	87.4	2.3±1.8	9.5
Mola07B	1.04	59	4.6	47.4	117.5	0.6	2.4	5.8×10^5^	0.50	69	13.2±24.5	108.4	2.2±1.8	9.2
Mola07C	1.43	153	4.9	74.7	135.4	0.4	0.6	5.4×10^5^	0.31	50	22.1±20.3	79.3	4.9±3.3	13.6

Records during the first three minutes were excluded.

**Figure 2:**
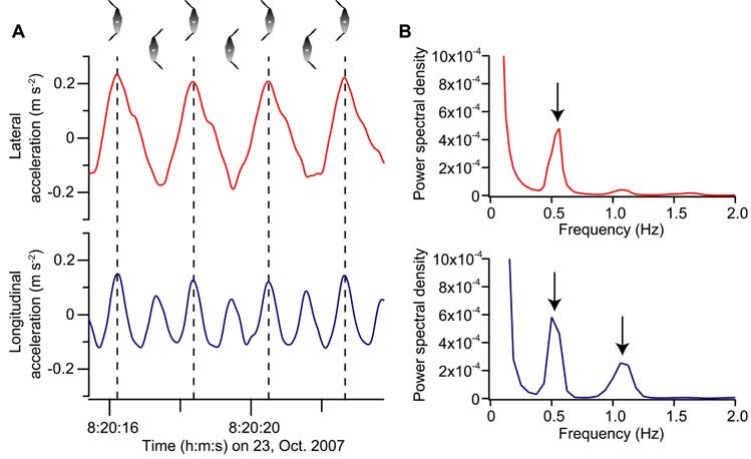
Lateral (red line) and longitudinal (blue line) accelerations recorded from an ocean sunfish (mass, 48 kg). (A) Typical examples. The vertical broken lines delineate the separation of the stroke cycle. (B) Power spectral density (PSD) calculated from the entire data set of this individual. Arrows indicate peaks.

Lateral acceleration oscillated regularly, showing that the fish stroked their dorsal and anal fins from side-to-side (Fig. 2A). Longitudinal acceleration also oscillated regularly and peaked twice during one stroke cycle (i.e., the period taken by a fin to move from one extreme lateral position and back to the original position), indicating that each fin stroke (left-to-right or right-to-left) produced thrust. The same results were shown when the power spectral density (PSD) was calculated from the entire data set of each individual. The PSD of lateral acceleration had one peak, while that of longitudinal acceleration had two peaks (Fig. 2B). The peak in lateral acceleration and the lower-frequency peak in longitudinal acceleration are the dominant stroke cycle frequency for each individual (Table 1). The higher-frequency peak in longitudinal acceleration represents the frequency at which thrust is produced (twice the dominant stroke cycle frequency).

### Morphological study

**Figure 3:**
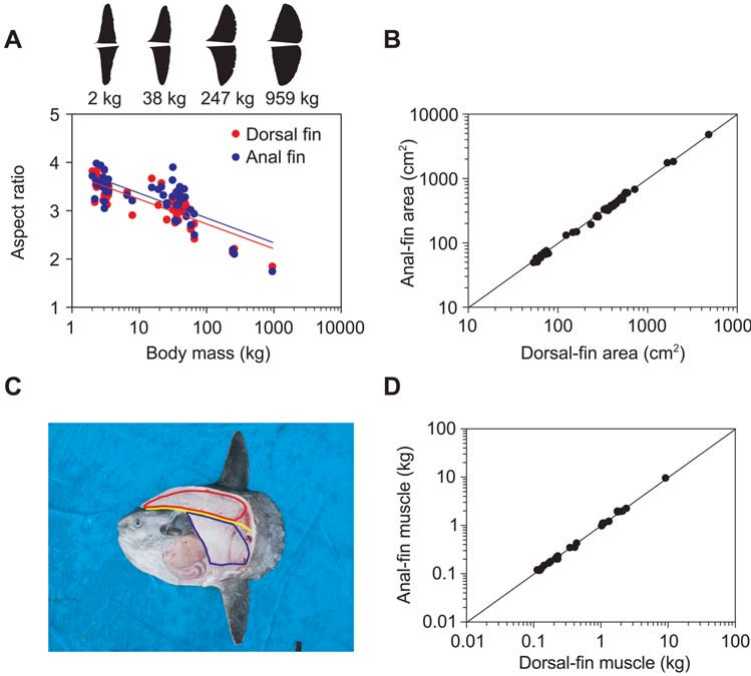
Morphology of locomotory fins and muscles of ocean sunfish. (A) Relationship between body mass and aspect ratio of dorsal fins (red circles) and anal fins (blue circles). The best-fit regression lines for dorsal (red line) and anal fin (blue line) are described as follows.
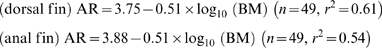
where AR is aspect ratio (dimensionless) and BM is body mass (kg). Examples of the shape of the fins (outlined from photos) from four mass groups are also shown. (B) Relationship between the projected area of dorsal and anal fins. The diagonal line represents identical area of both fins. (C) Photo of an individual (mass, 34 kg) showing how muscles that drive the dorsal fin (red line) and anal fin (blue line) are located. Yellow line indicates the vertebral column. (D) Relationship between the mass of dorsal-fin muscle and anal-fin muscle. The diagonal line represents identical mass of both muscles.

The two-dimensional shape of dorsal and anal fins of ocean sunfish changed with an increase in body mass from 2 kg to 959 kg, but symmetry in the shape between the two fins was maintained (Fig. 3A,B). Small fish (2 kg) had narrow fins with a shape similar to a tall isosceles triangle. As body mass increased, the fins became wider and the posterior edge of the fins became curved. Accordingly, aspect ratio (i.e., the ratio of length to average width) of both fins decreased significantly (*P*<0.0001 for both fins, *n* = 49) from an average of 3.6±0.2 for 2-kg individuals (*n* = 6) to 1.8 for a 959-kg individual (Fig. 3A). Dorsal and anal fins had the same projected area, with a concordance correlation coefficient [13] of 0.999 (*n* = 49) (Fig. 3B). Musculature of ocean sunfish is unusual for fishes. Axial musculature is entirely lost, and most muscles attached to the vertebral column are those for operating the dorsal and anal fins [14]. The dorsal-fin muscle occupies most of the space dorsal to the vertebral column under the subcutaneous gelatinous layer and skin, extending from the head to the anterior edge of the caudal-fin-like structure (Fig. 3C). In contrast, the anal-fin muscle occupies only the posterior part of the space ventral to the vertebral column because of the abdominal cavity. Despite the difference in shape, the mass of the two muscles was the same, with a concordance correlation coefficient of 0.999 (*n* = 24) in sunfish ranging in size from 2 kg to 247 kg (Fig. 3D).

Contrary to the hypothesis that ocean sunfish are negatively buoyant and swim continuously to avoid sinking, they were neutrally buoyant with a mean body density of 1,027±4 kg m^−3^ (*n* = 20), which is similar to that of seawater (c.a., 1,026 kg m^−3^). How do they attain neutral buoyancy without a swimbladder? Liver density significantly decreased with body mass (*P*<0.0005, *n* = 17), from an average of 1,041±9 kg m^−3^ for 2-kg individuals (*n* = 4) to 992 kg m^−3^ for a 247-kg individual, indicating an accumulation of lipid in the liver with growth. This means that the liver can act as a float in larger individuals. However, this effect should be minor because liver mass was only 2.6±0.9% (*n* = 22) of body mass on average, which is much less than 17–30% reported in some sharks [12]. Besides liver, most components of the body (e.g., skin, muscle, bone, and intestine) were denser than seawater as is the case in fishes generally [15]. However, we found that a subcutaneous gelatinous tissue plays a major role in making ocean sunfish neutrally buoyant. The subcutaneous gelatinous layer, which is an unusual feature of ocean sunfish, surrounded the whole body and became significantly thicker with body size (*P*<0.0001, *n* = 19), from an average of 3.9±0.8 cm for 2-kg individuals (*n* = 4) to 21.0 cm for a 959-kg individual at the belly just anterior to the cloaca. Furthermore, the caudal-fin-like structure was mostly made of the gelatinous tissue. The tissue was less dense (mean, 1,015±3 kg m^−3^, *n* = 22) than seawater regardless of body size (*P* = 0.17). The proportion of the mass of the gelatinous tissue to body mass significantly increased with body size (*P*<0.0001, *n* = 21), from an average of 26±6% for 2-kg individuals (*n* = 4) to 44% for a 247-kg individual. From the density and mass of gelatinous tissue and liver, we calculated that 69–100% of the weight in water was supported by the gelatinous tissue, whereas the rest was supported by the liver depending on body size.

## Discussion

Contrary to the common view that ocean sunfish are planktonic fish at the mercy of oceanic currents, the sunfish tagged in this study swam actively with continuous strokes of their fins while making substantial vertical movements in the water column (Table 1, Fig. 1). The cruising speeds of migrating fishes that were measured previously by indirect methods, including conventional tagging and tracking with acoustic devices, are highly variable (refer to Table 1 in [16]). However, recent studies that directly measured the swimming speed of fishes with animal-borne speedometers revealed that sturgeons [9], salmons [17], [18], marlins [19], and blue sharks [20] cruise at a relatively narrow range of speed (0.1–1.0 m s^−1^). The cruising speeds recorded in this study (0.4–0.7 m s^−1^) lie in the middle of the range, indicating that ocean sunfish are not slow swimmers. Although our sample size is small, an interesting observation is that larger fish swam slower, resulting in relatively constant Reynolds number (6×10^5^) among individuals (Table 1). Around this Reynolds number, drag on a streamlined body drops due to a transition of flow pattern [21]. Therefore, the speed recorded in this study might be their optimal speed that minimizes the cost required to travel a unit distance [22].

The acceleration data revealed that thrust was produced twice during one stroke cycle (Fig. 2), demonstrating that the thrust was lift-based rather than drag-based. This pattern of acceleration was previously reported in penguins [7], which flap both flippers synchronously, indicating that ocean sunfish oscillate the dorsal and anal fins synchronously. While the flippers of penguins are located in the coronal plane and oscillated dorsoventrally, the fins of ocean sunfish are located in the sagittal plane and oscillated laterally. In most animals, symmetrical wings for lift generation are located on both sides of the body, such as those found in flying insects, flying birds, and bats in air, and sea butterflies, eagle rays, sea turtles, penguins, and sea lions in water. The ocean sunfish is, to our knowledge, the only animal that uses two fins (i.e., the dorsal and anal fin) that are not originally bilaterally symmetrical as a pair of wings.

The dorsal and anal fins in each individual fish had symmetrical shape and identical area, although the shape of the fins changed with body size (Fig. 3A,B). This suggests that thrust generated by the two fins during a stroke cycle are equal, because lift produced by a wing is proportional to the area of the wing [5]. Although shape of muscles that drive the dorsal and anal fin differed markedly, the mass of the muscles were identical (Fig. 3C,D). This suggests that the dorsal and anal fins are driven with equal power. Dorsoventral symmetry in the external morphology of locomotor fins is echoed in the internal musculature that drive the fins. We suggest that this unusual symmetry represents a morphological adaptation for their unique swimming style.

A remarkable decrease in the aspect ratio of the dorsal and anal fins with growth (Fig. 3A) seems peculiar, because it indicates a decrease in the swimming efficiency with growth. Generally, wings with lower aspect ratio produce less lift and suffer more drag due to tip vortices, resulting in lower efficiency in producing thrust [5]. We hypothesize that the requirement of mechanical strength rather than efficiency shapes the fins in larger sunfish. Because the bending moment at the fin bases is proportional to the length of the fins, larger sunfish may need relatively shorter and wider fins to resist the moment while maintaining the area to generate sufficient lift. The theories of aeronautical engineering might be helpful in testing this hypothesis in future analyses.

We found that ocean sunfish are neutrally buoyant without a swimbladder because of their low-density subcutaneous gelatinous tissue. A similar strategy for attaining neutral buoyancy using gelatinous tissue was previously reported in some deep-sea fishes [23]. The advantage of gelatinous tissue over a swimbladder as a buoyancy aid would be that the former is not compressed by hydrostatic pressure and gives stable buoyancy regardless of depths. Indeed, fish with a neutral buoyancy due to a swimbladder experience changes in buoyancy at different depths, which affect their stroke patterns and body inclination [9]. The advantage of incompressible gelatinous tissue was clearly shown by our behavioral data. Our sunfish frequently descended and ascended in the water column while maintaining their own stroke cycle frequencies (Fig. 1), which are presumably optimal for their muscle characteristics. Furthermore, our observation that the sunfish occasionally swam in rotated positions is probably associated with their neutral buoyancy.

Other Tetraodontiform fishes are categorized as drag-based swimmers [24], which use a complex combination of the pectoral, dorsal, anal, and caudal fins (puffers [25], boxfish [26], and burrfish [27]) or undulations of the dorsal, anal, and caudal fins (triggerfish [28]). We suggest that ocean sunfish acquired a lift-based swimming mode by evolving a symmetrical pair of narrow dorsal and anal fins, and identical muscle mass for each fin. This style of locomotion is advantageous for cruising because it is mechanically more efficient [29] and gives more thrust per stroke at high speed [5] compared to drag-based swimming. This, together with neutral buoyancy due to incompressible gelatinous tissue, likely enables ocean sunfish to travel efficiently over long distances both horizontally and vertically despite their unusual shape.

## Materials and Methods

### Behavioral study

Three ocean sunfish were caught alive by set nets in Otsuchi Bay, Japan (39.4°N, 142.0°E). On the fishermen's boat, we pierced a tiny hole on the back of the fish, anterior to the dorsal fin, through the skin and the subcutaneous gelatinous layer. We attached a 128 Mbit W380L-PD2GT data logger (21 mm in diameter, 117 mm in length, 60 g in air; Little Leonardo Co., Tokyo, Japan) with devices for data recovery (time-scheduled releasing mechanism, float, and VHF transmitter) [8], [9] to the fish using the hole, and released the fish within five minutes. During the attachment procedure, we flushed the gills with fresh sea water. The total weight of the instruments was 144 g in air (0.1–0.3% of the body mass of the fish), and its buoyancy was offset 37 g in water. After the logger was detached from the fish, it was located with VHF radio signals and recovered by R/V ‘Yayoi’ from the International Coastal Research Center. Body mass of the fish was estimated from total length using the equation described below. Spectrogram [30] and power spectral density [7] of acceleration data were calculated as described previously. Before calculating spectrogram, we filtered lateral acceleration records to remove low-frequency signals that were assumed to be the result of various turning and rolling movements by the fish [31]. Dominant stroke cycle frequencies at each five-minute time bin (Fig. 1) were calculated from the spectrogram using a custom-written program in Igor Pro (WaveMetrics Inc., Lake Oswego, OR, USA). Because the fish's body yaws while swimming, thrusts of fish affect lateral acceleration records as well as longitudinal ones. Consequently, thrust frequency (twice stroke cycle frequency) rather than stroke cycle frequency was sometimes calculated as the dominant frequency from the spectrogram of lateral acceleration. We excluded the calculated values in this case, and therefore, dominant stroke cycle frequency in Fig. 1 has blanks. Pitch and roll was calculated from low-frequency signals of acceleration records [32]. Reynolds number was calculated as *LU*/*ν*, where *L* is total length of the fish (m), *U* is swimming speed (m s^−1^), and *ν* is kinematic viscosity of seawater at 17°C (1.13×10^−6^ m^2^ s^−1^). This research was conducted with a permit from the University of Tokyo (UT: 005).

### Fish used in morphological study

A total of 49 ocean sunfish caught in Otsuchi and Funakoshi Bay were collected at local fish markets. To weigh the fish, we used an electric platform scale for small individuals (less than 20 kg), a spring balance for middle-sized individuals (between 20 and 40 kg), and the electric gauge instrumented with the forklift at the markets for large individuals (more than 40 kg). Two fish including the largest individual were obtained after their muscles had been removed for selling. To estimate the mass of these fish, we added the published data of a very large individual [33] to our data sets to obtain the relationship between total length (TL, in m) and body mass (BM, in kg) for fish ranging in mass from 2 to 1,150 kg.
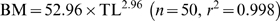



### Fin morphology and muscle mass

We took digital photos of fins of the all 49 fish (mass, 2–959 kg) with a reference square of known area, and counted pixels of the fins and the square with Igor Pro to calculate projected fin area. Because the base of the fins was not obvious in the photos, we marked the bending points of fins on the anterior and posterior edges before taking photos, and assumed that the base is the straight line segment between the two points. The relationships between the projected fin area (FA, in m^2^) and total length (TL, in m) for fish ranging in mass from 2 to 959 kg were as follows.
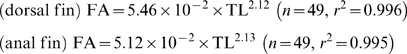



Fin length was measured as the minimum length between the base line and the tip of the fins. Aspect ratio was calculated as length squared divided by projected area. Locomotory muscles located on one side of the bodies (right or left) of 24 fish (mass, 2–247 kg) were dissected and weighed.

### Buoyancy

We used 22 fish (mass, 2–247 kg) to study the buoyancy of ocean sunfish. Because we did not measure some parameters (e.g., liver density) at the beginning of the study, the sample size ranged from 17 to 22 depending on the parameters. Density of whole body, gelatinous tissue, and liver was measured. When the materials floated in seawater and sank in freshwater, we mixed seawater and freshwater until they became neutrally buoyant, before measuring the water density (equivalent to the density of the materials) with a gravimeter. When they sank in seawater, we added salt to seawater until the materials became neutrally buoyant, before measuring the water density. Among our materials, only some livers floated in freshwater. In this case, we put small metal nails into the liver one by one until the liver became neutrally buoyant in freshwater (density, 1,000 kg m^−3^). The density of the liver was calculated from the mass of liver, mass of nails, and density of nails (7,780 kg m^−3^). The gelatinous tissue is strongly attached to the skin. To estimate the mass of gelatinous tissue, we cut it out and weighed it with the skin, and subtracted the skin mass from the compound mass. To estimate the skin mass, we first measured the surface area of the fish. This was done by covering the fish with a polyester sheet, tracing the midline of the fish with ink, and taking digital photos of the midline with a reference square of a known area, just as we did for fins. The area inside of the midline was doubled to obtain the total surface area. The relationship between the total length (TL, in m) and total surface area (SA, in m^2^) for fish ranging in mass from 2 to 247 kg was as follows.
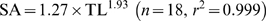



The surface areas of the dorsal and anal fins (i.e., twice the projected area of the fins measured from the photos) were subtracted from the total surface area, and the remaining area was multiplied by the skin mass of a unit area, which was measured directly, to obtain the skin mass.

### Statistical analysis

Spearman's rank correlation was used to test the correlation between body mass and parameters (e.g., liver density). Values for statistical significance were set at *P*<0.05. Means (±S.D.) are reported.

## References

[1] Johnson GD, Britz R (2005). Leis's conundrum: homology of the clavus of the ocean sunfishes. 2. Ontogeny of the median fins and axial skeleton of *Ranzania laevis* (Teleostei, Tetraodontiformes, Molidae).. J Morphol.

[2] McCann C (1961). The sunfish, *Mola mola*, in New Zealand waters.. Rec Domin Mus.

[3] Alexander RM (1967). Functional design in fishes.

[4] Webb PW (1984). Body form, locomotion and foraging in aquatic vertebrates.. Am Zool.

[5] Vogel S (1994). Life in moving fluids: The physical biology of flow. 2nd edn.

[6] Cartamil DP, Lowe CG (2004). Diel movement patterns of ocean sunfish *Mola mola* off southern California.. Mar Ecol Prog Ser.

[7] Sato K, Watanuki Y, Takahashi A, Miller PJO, Tanaka H (2007). Stroke frequency, but not swimming speed, is related to body size in free-ranging seabirds, pinnipeds and cetaceans.. Proc R Soc B.

[8] Watanabe Y, Baranov EA, Sato K, Naito Y, Miyazaki N (2004). Foraging tactics of Baikal seals differ between day and night.. Mar Ecol Prog Ser.

[9] Watanabe Y, Wei Q, Yang D, Chen X, Du H (2008). Swimming behavior in relation to buoyancy in an open swimbladder fish, the Chinese sturgeon.. J Zool.

[10] Nelson JS (1994). Fishes of the world. 3rd edn.

[11] Magnuson J (1970). Hydrostatic equilibrium of *Euthynnus affinis*, a pelagic teleost without a gas bladder.. Copeia.

[12] Corner EDS, Denton EJ, Forster GR (1969). On the buoyancy of some deep-sea sharks.. Proc R Soc B.

[13] Zar JH (1999). Biostatistical analysis. 4th edn.

[14] Gregory WK, Raven HC (1934). Notes on the anatomy and relationship of the ocean sunfish (*Mola mola*).. Copeia.

[15] Alexander RM (2003). Principles of animal locomotion.

[16] Beamish FWH, Hoar WS, Randall DJ (1978). Swimming capacity.. Fish physiology VII Locomotion.

[17] Tanaka H, Takagi Y, Naito Y (2001). Swimming speeds and buoyancy compensation of migrating adult chum salmon *Oncorhynchus keta* revealed by speed/depth/acceleration data logger.. J Exp Biol.

[18] Tanaka H, Naito Y, Davis ND, Urawa S, Ueda H (2005). First record of the at-sea swimming speed of a Pacific salmon during its oceanic migration.. Mar Ecol Prog Ser.

[19] Block BA, Booth D, Carey FG (1992). Direct measurement of swimming speeds and depth of blue marlin.. J Exp Biol.

[20] Carey FG, Scharold JV, Kalmijn AJ (1990). Movements of blue sharks (*Prionace glauca*) in depth and course.. Mar Biol.

[21] Hoerner SF (1965). Fluid dynamic drag: Practical information on aerodynamic drag and hydrodynamic resistance.

[22] Weihs D (1973). Optimal fish cruising speed.. Nature.

[23] Yancey PH, Lawrence-Berrey R, Douglas MD (1989). Adaptations in mesopelagic fishes. Buoyant glucosaminoglycan layers in species without diel vertical migrations.. Mar Biol.

[24] Webb PW, Evans DH (1993). Swimming.. The physiology of fishes.

[25] Gordon MS, Plaut I, Kim D (1996). How pufferfish (Teleostei: Tetraodontidae) swim.. J Fish Biol.

[26] Hove JR, O'Bryan LM, Gordon MS, Webb PW, Weihs D (2001). Boxfishes (Teleostei: Ostraciidae) as a model system for fishes swimming with many fins: kinematics.. J Exp Biol.

[27] Arreola VI, Westneat MW (1996). Mechanics of propulsion by multiple fins: kinematics of aquatic locomotion in the burrfish (*Chilomycterus schoepfi*).. Proc R Soc B.

[28] Blake RW (1978). On balistiform locomotion.. J Mar Biol Ass UK.

[29] Walker JA, Westneat MW (2000). Mechanical performance of aquatic rowing and flying.. Proc R Soc B.

[30] Sato K, Daunt F, Watanuki Y, Takahashi A, Wanless S (2008). A new method to quantify prey acquisition in diving seabirds using wing stroke frequency.. J Exp Biol.

[31] Sato K, Mitani Y, Cameron MF, Siniff DB, Naito Y (2003). Factors affecting stroking patterns and body angle in diving Weddell seals under natural conditions.. J Exp Biol.

[32] Johnson MP, Tyack PL (2003). A digital acoustic recording tag for measuring the response of wild marine mammals to sound.. IEEE J Ocean Eng.

[33] Kawakami Y, Hirao K, Ichisawa K, Ando S (2005). A record of the ocean sunfish, *Mola mola* (Tetraodontiformes: Molidae) from Shimane Prefecture, the Sea of Japan. (in Japanese).. Bull Tottori Pref Mus.

